# The usefulness of the Korean version of modified Mini-Mental State Examination (K-mMMSE) for dementia screening in community dwelling elderly people

**DOI:** 10.1186/1471-2458-4-31

**Published:** 2004-07-30

**Authors:** Seul-Ki Jeong, Ki-Hyun Cho, Jae-Min Kim

**Affiliations:** 1 Department of Neurology, Seonam University School of Medicine, 120-1, Mareuk-dong, Seo-gu, Gwangju, South Korea; 2 Department of Neurology, Chonnam University School of Medicine, South Korea; 3 Department of Psychiatry, Chosun University School of Medicine, South Korea

## Abstract

**Background:**

We assessed whether the Korean version of modified Mini-Mental State Examination (K-mMMSE) has improved performance as a screening test for cognitive impairment or dementia in a general population compared with the Korean Mini-Mental State Examination (K-MMSE).

**Methods:**

Screening interviews were conducted with people aged 65 and over in Noam-dong, Namwon-city, Jeonbuk province. There were 522 community participants, of whom 235 underwent clinical and neuropsychological examination for diagnosis of dementia and Cognitive Impairment No Dementia (CIND). Sensitivity, specificity and areas under the receiver operating characteristic (ROC) curves for the K-mMMSE and the K-MMSE were the main outcome measures.

**Results:**

Cronbach's alpha for the K-mMMSE was 0.91, compared with 0.84 for the K-MMSE. The areas under the ROC curves in identifying all levels of CIND or dementia were 0.91 for the K-mMMSE and 0.89 for the K-MMSE (*P *< 0.05). For the K-mMMSE, the optimal cut-off score for a diagnosis of CIND was 69/70, which had a sensitivity of 0.86 and a specificity of 0.79, while, for a diagnosis of dementia, the optimal cut-off score of 59/60 had a sensitivity of 0.91 and a specificity of 0.78. The K-mMMSE also had a high test-retest reliability (*r *= 0.89).

**Conclusion:**

Our findings indicate that the K-mMMSE is more reliable and valid than the K-MMSE as a cognitive screen in a population based study of dementia. Considering the test characteristics, the K-MMSE and modified version are expected to be optimally used in clinical and epidemiologic fields.

## Background

The Mini-Mental State Examination (MMSE) is a brief screening test that quantitatively assesses the cognitive status of elderly people [1,2]. It is easy to administer and has shown good reliability. Although its validity as a screening test is acceptable for clinical samples, it has been shown to have difficulty in discriminating between demented and non-demented individuals in community-based samples [2]. The MMSE has been found to be influenced largely by pre-morbid ability and is less sensitive to focal brain dysfunction [3] or mild dementia [4].

These limitations led to the development of the Modified Mini-Mental State Examination (3MS) in 1987 [5], which expanded the MMSE from 30 to 100 points to provide finer discrimination. In addition, the 3MS added four items: personal information, including date and place of birth; verbal fluency; abstract reasoning; and a second delayed recall trial. The 3MS also graded temporal orientation and broadened the delayed recall measures, which included cued and recognition formats. While retaining the brevity and ease of administration of the MMSE, the 3MS improved the validity and reliability of identifying individuals with dementia [6,7], and in predicting functional outcomes in patients with stroke [8].

In 2002, a group from the Cache County Study modified the 3MS for use as a cognitive screen in an epidemiologic study of dementia [9]. These modifications substituted the recall of personal demographic information with the recall of current and past prominent politicians. The main reason for this modification was the difficulty the researchers had in verifying personal demographic information. In addition, the scaling of the items in the time orientation and writing parts of the test was changed, and the time allotted for animal naming was shortened. This revised form of the 3MS (3MS-R) demonstrated good sensitivity in detecting dementia in a general population and providing age- and education-specific normative data and cut-off values at the 7^th ^and 10^th ^percentiles [9].

In Korea, the MMSE was translated into two versions, the Korean version of the Mini-Mental State Examination (MMSE-K) and the Korean Mini-Mental State Examination (K-MMSE) [10,11]. Both Korean versions of the MMSE were tested for validity and efficacy in clinical settings [11,12] and partly in epidemiologic research [13]. Both were somewhat modified to adjust better to the cultural background in Korea, but both shared all the limitations of the original MMSE. Recently the Korean Modified Mini-Mental State Examination (K-3MS) was introduced and validated in a clinical setting [14]. Although the K-3MS was found to be a reliable cognitive screening measure, there was no significant difference between the K-3MS and extracted MMSE for detecting individuals with dementia. In addition, components of the K-3MS could not be compared with items extracted from the K-MMSE and MMSE-K. We have therefore introduced the Korean version of modified Mini-Mental State Examination (K-mMMSE), and we have determined whether its validity is superior to that of the K-MMSE as a screen for cognitive impairment or dementia in a community setting.

## Methods

### Subjects

Potential participants for this study were recruited from all inhabitants of Noam-dong, Namwon-city, Jeonbuk-province, South Korea, aged 65 and over in 2003, as recorded in national residents registration lists. The area surveyed covered 8.93 km^2 ^and had an estimated population of 6,883, of whom about 7% were aged 65 or over. All participants gave informed consent, and the study was conducted in accordance with the guidelines in The Declaration of Helsinki and approved by the appropriate research ethics committee.

Among the 522 eligible subjects aged 65 and over identified from registration lists, 235 (45%) completed clinical examinations after the interview and formed the study sample for principal analysis. Of the remaining subjects, we were unable to establish contact with 162 (31%), 75 (14%) refused to participate, 18 (3%) did not complete the survey, 7 (1%) had severe pre-morbid illness including blindness and deafness, 4 (0.8%) had changed address, and 3 (0.6%) had died before the visit. The principal apparent reason for the difficulties in establishing contact was that the person was in a regular daily activity or away from home, visiting family members living elsewhere. The 18 individuals (3%) who did not complete the survey questionnaire or were not examined clinically had a mean age of 74.9 ± 10.3 years; 13 (72%) were females, and 4 (22%) were educated. Of all the eligible subjects, participants and non-participants did not differ in age (73.5 ± 6.8 y vs. 74.6 ± 7.8 y, respectively) or gender (66% and 62%, all *P *values > 0.1).

### Assessment and measurements

Interviewers received a seven-day training session on administering the screening instruments and were supervised throughout by the project neurologist. Cognitive status was classified in two stages. In the first stage, interviewers carried out home-based interviews for data on cognitive function, past medical history, and demographic characteristics. All participants were contacted for cognitive screening using a formulated battery, from which the K-mMMSE and K-MMSE were extracted. And they were rated by a knowledgeable informant using the Short form of Samsung Dementia Questionnaire (S-SDQ) and Korean Instrumental Activities of Daily Living (K-IADL) [15,16]. The S-SDQ is a Korean version of the Informant Questionnaire on Cognitive Decline in the Elderly (IQCODE), with 15 items and scores ranging from 0 to 30 [15]. The K-IADL is composed of 11 items that grade functional abilities, with scores calculated as the sum of points over the number of applicable questions and ranging from 0.0 to 3.0 [16]. High scores on the S-SDQ and K-IADL indicate poor performance. Both tests were found to be uncontaminated by pre-morbid ability, including education or age [15,16].

At the second interview, physicians who were blinded to the cognitive scores performed a clinical examination and neuropsychiatric inventory on participants who completed the first survey questionnaire. The clinical examination verified the presence of cognitive impairment. The diagnostic criteria for dementia were based on those of the Diagnostic and Statistical Manual of Mental Disorders (DSM-IV) [17] and were subdivided according to the guidelines of the National Institutes of Neurological and Communicative Disorders and Stroke and the Alzheimer's disease and Related Disorders Association (NINCDS-ADRDA) [18]. Severity of dementia was staged using the Korean version of the Expanded Clinical Dementia Rating (CDR) scale [19]. The physician and neurologist made independent diagnoses and CDR scoring and subsequently held a case conference to reach a consensus diagnosis, classifying the person as either cognitively normal, cognitively impaired with no dementia (CIND), or having dementia. A diagnosis of CIND represents an attempt to classify people with recognizable cognitive decline who did not meet the criteria for dementia. This group included people who complained of cognitive decline and showed impaired memory function, but did not have any non-cognitive alterations, including intact activities of daily living. At both stages, home visits were repeated on at least two occasions if no contact was made.

### Instruments

We translated the original 3MS-R into Korean according to the guidelines recommended by the modifiers [9]. The item regarding political figures, which asked the participants to name the current president, vice president, and state governors, was replaced with questions about current and previous presidents, because there is no vice president in the government of South Korea. We assessed temporal orientation according to three methods used to calculate year and time in Korea: the solar, lunar and Tangun era. We replaced the words "shirt," "nickel" and "honesty" in the memory task with the words "airplane," "pine tree" and "sincerity." In Korean, the first two words coincided with the K-MMSE items and ended with vowel sounds ("bee-haeng-gi," and "so-na-mu," respectively); while "seong-sil" in Korean, which means sincerity, was substituted for "jeong-jik," which is equivalent to the English word honesty, inasmuch as a pilot study found that "jeong-jik" was more difficult to hear or perceive than "seong-sil", possibly because the latter ended with a voiced sound and could be heard more comfortably.

In explaining the appropriate questions and answers, we presented a simple example prior to asking the first question, specifically, "the eyes and nose are different, but they are similar in being part of our face." In a pilot study, most elderly subjects could not understand the concept of similarity without this example, and they became intolerant to our interview unless an example was provided. After providing the example, however, most subjects were more cooperative and tried to answer properly. We asked subjects to write a spontaneous sentence and scored whether it was legible and correct, with or without prompting. The total possible score was 100, and a K-MMSE score could be generated from it.

### Statistical analysis

For demographic factors, mean or median values and proportions were calculated according to the cognitive impairment strata. Cronbach's alpha was used to quantify internal consistencies of the K-mMMSE and K-MMSE [20]. We assessed the stability and test-retest reliability with 30 subjects who took the K-mMMSE twice. Receiver operating characteristic (ROC) curves were used to determine the validity of the two screening tests graphically and statistically. The areas under the ROC curves (AUC) were calculated and described with standard errors (SE) using the trapezoidal rule [21], and comparison between AUCs was made by the algorithm with an estimated covariance matrix [22]. Cut-off points were chosen to optimize the trade-off between false-negative and false-positive rates. The choice of whether to judge the screening tests by their ability to identify CIND or dementia was addressed practically by assessing both. The validity of the K-mMMSE and K-MMSE were compared, first between those with CIND or dementia and those who were normal, and then between those with dementia and those with CIND or normal. All statistical calculations were performed using Stata 8.2 software (Stata, College Station, TX).

## Results

### Descriptive statistics

Descriptive statistics are displayed in Table 1 for the total sample and according to the cognitive impairment strata. Of the 235 participants, 46 (19.6%) were classified as having dementia and 54 (22.9%) as having CIND. Overall levels of education were very low, in that 118 participants (50.2%) had no formal education, of whom 83 (70.3%) were illiterate.

Severity of cognitive impairment was directly related to mean age and inversely related to number of years of education. Women had higher rates of being cognitively impaired or demented than men. The median scores on the K-mMMSE and K-MMSE decreased with severity of cognitive impairment. Median scores on the informant questionnaires of the S-SDQ and K-IADL increased with poorer cognitive status. The inter-quartile ranges (IQR) also steadily increased, reflecting increasing variability in cognitive and functional status. Four respondents scored perfectly on the K-MMSE, whereas none scored perfectly on the K-mMMSE, suggesting that the K-mMMSE might be less prone to the ceiling effect in this population.

**Table 1:** Subject characteristics

	All subjects (n = 235)	Normal (n = 135)	CIND (n = 54)	Dementia (n = 46)
**Demographic characteristics**				

Age, mean ± SD, y	73.5 ± 6.7	71.9 ± 5.3	73.8 ± 7.4	77.8 ± 7.8
Women, %	66.4	57.8	74.1	82.6
Education, median (IQR), y	1 (0–6)	4 (0–6)	0 (0–5)	0 (0–2)

**Cognitive measures, median (IQR), score**				

K-mMMSE	64 (48–80)	78 (66–85)	54 (44–63)	38 (29–47)
K-MMSE	20 (14–25)	24 (20–27)	16 (13–21)	12 (9–15)
S-SDQ	9 (5–13)	7 (3–10)	10 (5–14)	14 (10–21)
K-IADL	0.22 (0.09–0.50)	0.11 (0.00–0.29)	0.27 (0.11–0.60)	1.10 (0.40–1.67)

CIND; Cognitive Impairment No Dementia, SD; standard deviation, IQR; Inter-quartile range, K-mMMSE; Korean version of modified Mini-Mental State Examination, K-MMSE; Korean Mini-Mental State Examination, S-SDQ; Short form of the Samsung Dementia Questionnaire, K-IADL; Korean Instrumental Activities of Daily Living.

### Reliability

The estimated Cronbach's alpha was 0.91 for the K-mMMSE and 0.84 for the K-MMSE. Relative to each cognitive impairment stratum (normal, CIND, and dementia), the alphas were 0.84, 0.81 and 0.81, respectively, on the K-mMMSE and 0.74, 0.72, and 0.63, respectively, on the K-MMSE. Neither age nor gender had any substantial impact on internal consistency. Stratum-specific alphas of the K-mMMSE for the different subgroups ranged from 0.86 to 0.91 in men and 0.89 to 0.90 in women.

The retest of the K-mMMSE was assessed in 30 subjects (mean interval, 26 days; range, 19–32 days). The correlation coefficients for the total scores were 0.89 on the K-mMMSE and 0.85 on the K-MMSE. The coefficients of the 15 items of the K-mMMSE were all significant, ranging from 0.37 for similarities to 0.83 for time orientation. The re-tested subjects were representative of the entire study population, in that the sociodemographic characteristics of the 30 retested subjects were similar to the other participants in mean age (74.0 ± 7.4 y vs. 73.5 ± 6.7 y), educational years (3.5 ± 3.2 y vs. 3.4 ± 3.9 y), K-mMMSE scores (63.8 ± 21.0 vs. 63.1 ± 20.4), and proportion of women (80.0% vs. 66.4%; *P *= 0.132).

### Validity of the K-mMMSE and K-MMSE

#### Construct validity

Construct validity data between the K-mMMSE and other cognitive or functional measures are shown in Table 2. K-mMMSE was found to be significantly correlated with all measures, including CDR, Sum of Boxes of CDR (CDR-SB), and informant questionnaires such as the S-SDQ and K-IADL. The correlation coefficient between K-mMMSE and K-MMSE scores was 0.94. According to the CDR scores, the median values of the K-mMMSE and K-MMSE changed significantly (Table 3).

**Table 2:** Correlations between K-mMMSE, K-MMSE and cognitive or functional measures (CDR, KIADL, and S-SDQ)

	K-mMMSE	K-MMSE	CDR	CDR-SB	KIADL	S-SDQ
K-mMMSE	1.000					
K-MMSE	0.945*	1.000				
CDR	-0.755*	-0.710*	1.000			
CDR-SB	-0.750*	-0.702*	0.966*	1.000		
KIADL	-0.648*	-0.614*	0.740*	0.793*	1.000	
S-SDQ	-0.489*	-0.454*	0.529*	0.555*	0.617*	1.000

* *P *< 0.001 by Pearson's correlation analyses. K-mMMSE; Korean version of modified Mini-Mental State Examination, K-MMSE; Korean Mini-Mental State Examination, CDR; Clinical Dementia Rating, CDR-SB; Sum of Boxes of CDR, K-IADL; Korean Instrumental Activities of Daily Living, S-SDQ; Short form of the Samsung Dementia Questionnaire

**Table 3:** K-mMMSE and K-MMSE scores for each CDR group

	CDR			
	
	0 (n = 137)	0.5 (n = 52)	1 (n = 33)	2+ (n = 13)
K-mMMSE, Median (IQR)*	78 (66–85)	54 (44–63)	44 (35–50)	29 (12–31)
K-MMSE, Median (IQR)*	24 (20–27)	16 (13–21)	12 (9–16)	10 (5–11)

* *P *< 0.001 by Kruskal-Wallis test. K-mMMSE; Korean version of modified Mini-Mental State Examination, K-MMSE; Korean Mini-Mental State Examination, CDR; Clinical Dementia Rating, IQR; Inter-quartile ranges.

#### Identification of combined CIND and dementia

**Figure 1:**
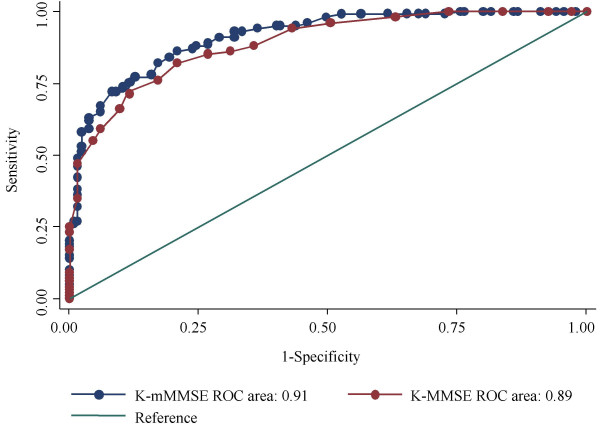
**Receiver operating characteristic (ROC) curves of the K-mMMSE and the K-MMSE for combined CIND and dementia. **K-mMMSE (light blue), K-MMSE (brown), and diagonal line. Area under ROC Curves (AUC): K-mMMSE = 0.91, K-MMSE = 0.89.

The performance of the K-mMMSE (AUC ± SE, 0.91 ± 0.02) was significantly superior to that of the K-MMSE (0.89 ± 0.02; *P *= 0.041). The ROC curves plotted in the same graph suggested that the performance of the K-mMMSE was superior to that of the K-MMSE at almost all cut-off points (Figure 1). At a cut-off of 69/70 for CIND, the K-mMMSE had a sensitivity of 0.86 (95% Confidence Intervals, 0.78–0.92), a specificity of 0.79 (0.71–0.86), a positive likelihood ratio (LR) of 4.15, and a negative LR of 0.18. In comparison, at a cut-off of 20/21 for CIND, the K-MMSE had a sensitivity of 0.82 (0.73–0.89), a specificity of 0.79 (0.71–0.86), a positive LR of 3.95 and a negative LR of 0.23.

#### Identification of dementia

Both the K-mMMSE and the K-MMSE could properly discriminate demented from normal individuals, but there was no significant difference between them (AUC, 0.92 vs 0.91; *P *= NS). At the cut-off of 59/60, the K-mMMSE had a sensitivity of 0.91 (0.79–0.98), a specificity of 0.78 (0.72–0.84), a positive LR of 4.21 and a negative LR of 0.11. At a cut-off point of 18/19, the K-MMSE had a sensitivity of 0.91 (0.79–0.98), a specificity of 0.76 (0.69–0.82), a positive LR of 3.82 and a negative LR of 0.11.

## Discussion

We have shown here that the K-mMMSE is a valid, reliable, and stable cognitive screening instrument, as well as being more sensitive to all levels of CIND and dementia, compared to the K-MMSE. The K-mMMSE has been shown to have a broader spectrum of cognitive domains, including political figures, word fluency, similarities, and delayed recall. Furthermore, the expanded 100 point scoring allows finer discrimination of cognitive impairment. Thus, the K-mMMSE represents a summary form of administration and scoring.

Internal consistency results of the K-mMMSE and K-MMSE were comparable to those observed in previous community studies. Cronbach's alpha (á) for the 3MS has been reported to be 0.91 in a community study, a value identical to that found here [23]. Another population study has reported alphas for the 3MS and MMSE of 0.87 and 0.78, respectively, which were slightly lower than our findings of 0.91 and 0.84 [6]. Cronbach's alpha has been reported to be influenced by educational status or variability of response, in that it was higher in groups having fewer years of education [24] and in clinical populations having greater variability [25]. Our population consisted of a high percentage with no formal education (50.2%), and their scores were very variable (inter-quartile ranges for the K-mMMSE and the K-MMSE of 48–80 and 14–25, respectively).

Test-retest reliability results of the K-mMMSE were also comparable to those in previous studies. Correlation coefficients of 0.91 to 0.93 have been reported for small samples of community dwelling residents and dementia patients, which are slightly higher than our value of 0.89 [26]. The Stirling County Study found a coefficient of 0.78, but items requiring less judgment exhibited lower reliability than items requiring more judgment [23]. In contrast, we found markedly lower reliability in items requiring more judgment, i.e., similarities (*r *= 0.37), compared with simple items, i.e., temporal orientation (*r *= 0.81). The discrepancy might be due to a difference of time lag, in that the Stirling County Study retest was performed over a 3 year interval, with individual retests ranging from 0.9 to 4.0 years. Furthermore their retested subjects were not representative of all participants. We observed a correlation coefficient of 0.85 for the K-MMSE over all levels of cognitive status, which is in line with generally acceptable findings [2]. Scores on the K-mMMSE and K-MMSE increased after retest, with differences in mean values of 4.4 and 2.6 points, respectively, presumably due to a practice or studying effect after a short interval [1,27,28].

The K-mMMSE was superior to the K-MMSE for diagnosis of all levels of CIND or dementia, as well as being slightly superior at almost all cut-off points. Since dementia is usually preceded by CIND or mild cognitive impairment (MCI), the definition of both requires explication [29,30]. Subjects with CIND or MCI have been found to be at increased risk for developing dementia or, more specifically, Alzheimer's disease and some vascular subtypes of dementia [30,31]. The difference between the K-mMMSE and the K-MMSE in diagnosing this condition might mean that the former was more sensitive to the mild stage or pre-dementia than the latter. In this respect, the K-mMMSE seemed to partially overcome a weakness of the K-MMSE, that is, insensitivity to mild dementia [4]. The present findings suggested that the K-MMSE was actually a fairly reasonable instrument as well. Given the faster administration of the K-MMSE, it would be a choice of clinicians to use optimally, recognizing that the K-MMSE was slightly inferior in terms of its test characteristics.

The two cognitive screening measures did not differ significantly, however, in the detection of dementia. These results are comparable to the findings of McDowell et al. [6]; however, their validity results differed between the two language groups studied, namely French and English speakers. The 3MS was superior only in the diagnosis of combined CIND or dementia in French, but not English, speaking participants. There were fewer French than English participants (434 vs. 1166), and they had fewer years of education (6.8 vs. 9.2 years). These differences were also observed in our study samples, with the most important being the smaller sample size, inasmuch as statistical significance was directly influenced by the total number of participants [22].

Even Cache County modifications to the 3MS showed a good sensitivity and specificity, 3MS-R was also dependent on their cultural and social factors which might limit general use in non-US population [9]. For this reason, cross validation was a very important step for a cultural validation of the instrument. The K-mMMSE was shown to be more significantly correlated with other tests for cognitive status or functional abilities, such as the CDR, S-SDQ, and K-IADL, than was the K-MMSE. The correlation coefficients of the CDR were higher than the informant questionnaires, which might be due to the characteristics of the questions. That is, the K-mMMSE and K-MMSE are cognitive screening measures, and the CDR includes items about the cognitive aspects for scoring, whereas the informant questionnaires (S-SDQ and K-IADL) are comprised only of questions related to functional abilities. To the best of our knowledge, this is the first report showing concurrent validity of the modified MMSE series.

There are important limitations to our findings. First, the subjects who participated in this study showed very low levels of educational background, perhaps limiting its general usefulness, especially regarding the cut-off points for a diagnosis of CIND or dementia. The low educational attainment, however, has been one of important characteristics of our elder population, because they were largely deprived of education due to Korean War and Japanese colonial dominion over the country [32]. And the study design, which showed the validities of and comparison between the two cognitive screening measures, would be appropriate for selected community samples, because all participants have a two-stage interview and a clinical examination, thus reducing verification bias. Second, although the trapezoidal rule provide a more accurate method of estimating the "true" AUC, an AUC derived from the parameters of a straight-line fit to the ROC plot tends to slightly underestimate the AUC of a Gaussian-based ROC. Finally, although we observed no significant differences between participants and non-participants, the rate of participation in our study was somewhat low. The majority of non-participants were those with whom we could not meet on two separate visits, suggesting that individuals who refused to participate may be more intelligent or active than the participants. If this were true, however, our results would not change, and additional statistical power may be added to our analysis.

## Conclusions

We conclude that the K-mMMSE is a valid, stable, and reliable cognitive screen in an epidemiologic study. The K-mMMSE is more sensitive to all levels of CIND and dementia than the K-MMSE. Future investigations with the K-mMMSE will examine age-, sex-, and education-specific reference values to determine how performance patterns on individual items may discriminate between those with or without cognitive impairment and dementia subtypes.

## Competing interests

None declared.

## Authors' contributions

SKJ performed physical measurements, collected data, and drafted the manuscript. KHC participated in data collection and reviewed the manuscript. JMK conceived of the study and participated in its design, and also performed physical measurements. All authors read and approved the final manuscript.

## Pre-publication history

The pre-publication history for this paper can be accessed here:


